# Effect of Cafeteria Diet History on Cue-, Pellet-Priming-, and Stress-Induced Reinstatement of Food Seeking in Female Rats

**DOI:** 10.1371/journal.pone.0102213

**Published:** 2014-07-15

**Authors:** Yu-Wei Chen, Kimberly A. Fiscella, Samuel Z. Bacharach, Donna J. Calu

**Affiliations:** Intramural Research Program, NIDA/NIH, Baltimore, Maryland, United States of America; Radboud University, Netherlands

## Abstract

**Background:**

Relapse to unhealthy eating habits is a major problem in human dietary treatment. The individuals most commonly seeking dietary treatment are overweight or obese women, yet the commonly used rat reinstatement model to study relapse to palatable food seeking during dieting primarily uses normal-weight male rats. To increase the clinical relevance of the relapse to palatable food seeking model, here we pre-expose female rats to a calorically-dense cafeteria diet in the home-cage to make them overweight prior to examining the effect of this diet history on cue-, pellet-priming- and footshock-induced reinstatement of food seeking.

**Methods:**

Post-natal day 32 female Long-Evans rats had seven weeks of home-cage access to either chow only or daily or intermittent cafeteria diet alongside chow. Next, they were trained to self-administer normally preferred 45 mg food pellets accompanied by a tone-light cue. After extinction, all rats were tested for reinstatement induced by discrete cue, pellet-priming, and intermittent footshock under extinction conditions.

**Results:**

Access to daily cafeteria diet and to a lesser degree access to intermittent cafeteria diet decreased food pellet self-administration compared to chow-only. Prior history of these cafeteria diets also reduced extinction responding, cue- and pellet-priming-induced reinstatement. In contrast, modest stress-induced reinstatement was only observed in rats with a history of daily cafeteria diet.

**Conclusion:**

A history of cafeteria diet does not increase the propensity for cue- and pellet-priming-induced relapse in the rat reinstatement model but does appear to make rats more susceptible to footshock stress-induced reinstatement.

## Introduction

Excessive consumption of unhealthy palatable foods is a major public health problem contributing to obesity and obesity-related diseases [1], [2]. While many people attempt to control their food intake through dieting, most relapse to maladaptive eating habits within a short time [3]–[6]. This relapse is often triggered by acute exposure to palatable food, food-associated cues, or stress [6]–[10]. In order to model this clinical situation, we and the others have adopted the rat reinstatement model [11], which has been used extensively to study relapse to drug seeking [12]–[14]. In this model, rats are trained to lever-press for palatable food. After extinction of the food-reinforced responding, the rats are tested for reinstatement induced by non-contingent exposure to palatable food, food-associated cues, or stressors [15], [16].

The reinstatement procedure, which is relatively simple to implement, has proven to be very suitable for studying relapse to palatable food seeking during dieting because it reliably induces robust reinstatement effects across a variety of reinstating stimuli [16]. Nevertheless, this reinstatement model fails to capture certain aspects of the clinical problem. First, the target population seeking treatment for relapse to unhealthy eating after dieting is primarily comprised of overweight and obese individuals, and the proportion of women taking dietary supplements or seeking dietary treatment is more than twice that of men [17], [18]. Yet most pre-clinical relapse studies to date use normal-weight male rats to study relapse to palatable food seeking [15], [19]. Second, in the reinstatement procedure rats are typically given limited access to nutritionally balanced chow during the training and extinction/testing phases in order to promote enhanced motivation in the operant chamber. While humans with unhealthy eating habits generally consume limited amounts of nutritionally balanced foods and greater amounts of calorically-dense palatable foods, during dieting, subjects not only restrict the amount of calorically-dense food they eat but also tend to consume nutritionally balanced foods. These aspects of the human situation are not accurately modeled using standard chow food deprivation throughout the reinstatement procedure.

To increase the clinical relevance of the relapse to palatable food seeking model, we gave young female rats daily or intermittent (Monday, Wednesday, Friday) access to a calorically-dense cafeteria diet in the home-cage prior to and throughout the training period in order to make the rats overweight. Exposure to highly palatable and energy-dense food not only leads to obesity in rodents, but also causes dysregulation of reward processing and stress responses [20]–[22], both of which implicated in relapse behavior [16], [23]. Therefore, to more accurately model human dieting, we terminated access to cafeteria diets at the end of the training phase and examined the effect of diet history on discrete cue-, pellet priming-, and footshock-induced reinstatement of food seeking after extinction.

## Materials and Methods

### Subjects and apparatus

Female Long-Evans (total N = 24) rats (Charles River Laboratories, Wilmington, MA; 26 days old at time of arrival) were maintained on a reverse 12 h light/dark cycle (lights off at 8 AM) and were given free access to standard laboratory chow and water during the experiment. Rats were individually housed on post-natal day (PND) 30 and were weight matched across groups before being assigned to each diet condition (n = 8 per group). Diet manipulations began on PND 32. Following 50 days of diet exposure, rats began behavioral training. We performed the experiments in accordance to the “Guide for the care and use of laboratory animals” (8th edition, 2011, US National Research Council) and experimental protocols were approved by the Intramural Research Program (NIDA) Animal Care and Use Committee.

Behavioral experiments were conducted in standard self-administration chambers (Med Associates). Each chamber had two levers 9 cm above the floor, but only one lever (the “active,” retractable lever) activated the pellet dispenser, which delivered 45 mg food pellets containing 12.7% fat, 66.7% carbohydrate, and 20.6% protein (catalog #1811155; Test Diet). This pellet type was chosen based on pellet preference tests in food-restricted female rats, using six pellet types (obtained from Test Diet and Bioserv) with different compositions of fat (0–35%) and carbohydrate (45–91% sugar pellets) and different flavors (no flavor, banana, chocolate, grape) [15]. Rats were housed in the animal facility and transferred to the self-administration chambers prior to the training sessions, and returned to the facility at the end of the 2-h sessions.

### Diet manipulations

The chow only rats received free access to standard laboratory chow (Teklad global 18% protein rodent diet, Harlan Laboratories) (58% carbohydrate, 24% protein, 18% fat).

The daily cafeteria diet rats also had free access to standard laboratory chow in addition to a rotating schedule of highly palatable “cafeteria items.” This diet consisted of two items per day—one savory item and one sweet item. Two weeks on this diet allowed for a full rotation of cafeteria diet items including potato chips, corn snacks, chocolate cake, peanut butter cookies, pepperoni, pretzels, chocolate sandwich cookies, chocolate-chip cookies, and breakfast pastries. Both the chow and the cafeteria items were pre-weighed and the savory and sweet items were placed in two separate ceramic ramekins in the rats' home-cages, while the chow was available in the home-cage food hopper. Twenty-four hours later any chow or cafeteria items remaining in the cage were independently weighed to determine daily intake for each item. Daily intake of cafeteria items and chow was monitored Monday through Thursday. The amount of each item consumed per rat was converted to calories (kcal) using data provided by the manufacturers. The intermittent cafeteria diet rats received the same rotation of highly palatable cafeteria items that the daily cafeteria rats received in addition to free access to standard laboratory chow. However, these rats had 24 h of forced abstinence from the cafeteria items every Tuesday, Thursday, Saturday, and Sunday, during which the chow was available. Using the identical procedure described above, daily intake of cafeteria items (Monday, Wednesday, Friday) and chow (every day) was monitored and the caloric intake (kcal) from each item was calculated, except that the consumption was monitored Monday through Saturday. This was due to the intermittent schedule of this diet, in which cafeteria items had to be removed on Saturday morning following the 24 h access that began Friday morning. All rats were weighed daily during home-cage diet exposure and before each training, extinction, and reinstatement testing session.

### Training, extinction and reinstatement of food-reinforced responding

The experimental conditions used during training, extinction and reinstatement phases were similar to those used in previous studies in male and female rats [19], [24], [25], with the exception of food restriction and duration of sessions.

### Training and extinction

Behavioral training began with 2 days of magazine training which involved 2-h sessions during which pellets (Test Diet Purified Rodent Tablet (5TUL)) were delivered non-contingently, every 5 min into a food cup located to the right of the active lever. Pellet delivery was accompanied by a compound 5-s tone (2900 Hz)-light (7.5W white light) cue, both located above the active lever. Subsequently, rats were trained to self-administer the pellets on a fixed-ratio-1 (FR-1), 20-s timeout reinforcement schedule. At the start of each 2-h session, the red house light was turned on and the active lever was extended. Reinforced active lever presses resulted in delivery of one pellet, accompanied by the compound 5-s tone-light cue. Active lever presses during the 20-s timeout or presses on the inactive lever had no programmed consequences. Rats underwent 10 training sessions under these conditions. During training, rats were maintained on their respective diets.

Following ten 2-h food self-administration training sessions, all rats underwent no-cue extinction sessions in which active lever presses were no longer reinforced with a food pellet or the tone-light cue. During the extinction phase, in order to mimic human dieting, daily cafeteria and intermittent cafeteria rats were switched to free access chow diet, no longer receiving access to the cafeteria items in the home-cage. Chow rats experienced no change in the free access chow home-cage diet condition.

All rats were given 11 extinction sessions (no cue), and reached an extinction criterion (mean active lever pressing across the last three extinction sessions <20% of extinction day 1 responding) before the cue-induced reinstatement test began. Before the pellet-priming- and footshock-induced reinstatement tests started, all rats underwent another 7 and 2 sessions, respectively, of 2-h extinction, in which responses on the previously active lever led to tone-light cue presentations but no pellets (extinction with cue). On the day of the pellet-priming or footshock-induced reinstatement test, all rats underwent additional within-session extinction, and the reinstatement manipulation started after an extinction criterion was reached (<20 active lever responses within 1 h).

### Cue-induced reinstatement

After all rats reached extinction criterion, they underwent one day of cue-induced reinstatement testing. Cue reinstatement testing consisted of a single 2-h session beginning with 1 non-contingent tone-light cue presentation immediately after the session started [26]–[28]. During the session, active lever presses resulted in tone-light cue but no pellet (20 s timeout). Active lever presses during the 20-s timeout or presses on the inactive lever had no programmed consequences.

### Pellet-priming-induced reinstatement

After 7 sessions of 2-h daily extinction (with cue) session, the rats underwent one day of within-session extinction/pellet priming-induced reinstatement testing. The pellet priming test consisted of six 60 min mini-sessions separated by 5 min time out, in which the active lever was retracted and the house light was extinguished. The first 3 mini-sessions were conducted under extinction conditions (with cue) with the third hour serving as the 0 pellet baseline mini-session. At the start of the remaining 3 mini-sessions 1, 2, or 4 pellets were delivered non-contingently (each was separated by 20 s) into the food hopper, in ascending order. The within-session priming procedure is based on previous studies with cocaine priming [29], [30].

### Footshock-induced reinstatement

The rats underwent 2 additional 2-h daily sessions of extinction with cue between pellet priming- and footshock-induced reinstatement tests. For the test, the rats underwent one day of within-session extinction/footshock-induced reinstatement testing, which consisted of five to six mini-sessions. The testing started with three to four 60 min extinction mini-sessions separated by 5 min time out, in which the active lever was retracted and the house light was extinguished. These mini-sessions were conducted under extinction conditions (with cue), with the third or fourth hour serving as the 0 min duration shock baseline. A 10 min time out in which the active lever was retracted and the house light was extinguished followed this baseline session. After this timeout, rats received 5 min of intermittent footshock (0.18–0.30 mA based on individual shock reactivity; 0.5 s ON and an average of 40 s OFF period, 10–70 s intershock interval) with house light extinguished and lever retracted. Subsequently, the effect of 5-min footshock on reinstatement was assessed in a 60-min session under extinction conditions. After another 10 min time out, they received 10 min of intermittent footshock (0.18–0.30 mA based on individual shock reactivity). Reinstatement effect was again assessed in a 60-min session post-footshock. The within-session footshock-induced reinstatement procedure is based on a previous study with heroin [31] and the shock ON and OFF duration is based on previous studies on footshock-induced reinstatement of drug seeking [32], [33].

### Statistical analyses

Data were analyzed using the SPSS statistical software (IBM). The factors used in the statistical analyses are described in the Results section below. Results are presented as group means (±SEM). Significant main effects and interaction effects (p<0.05) from ANOVAs were followed by post-hoc Fisher PLSD tests when appropriate.

## Results

### Effect of home-cage diet history on body weight gain

**Figure 1:**
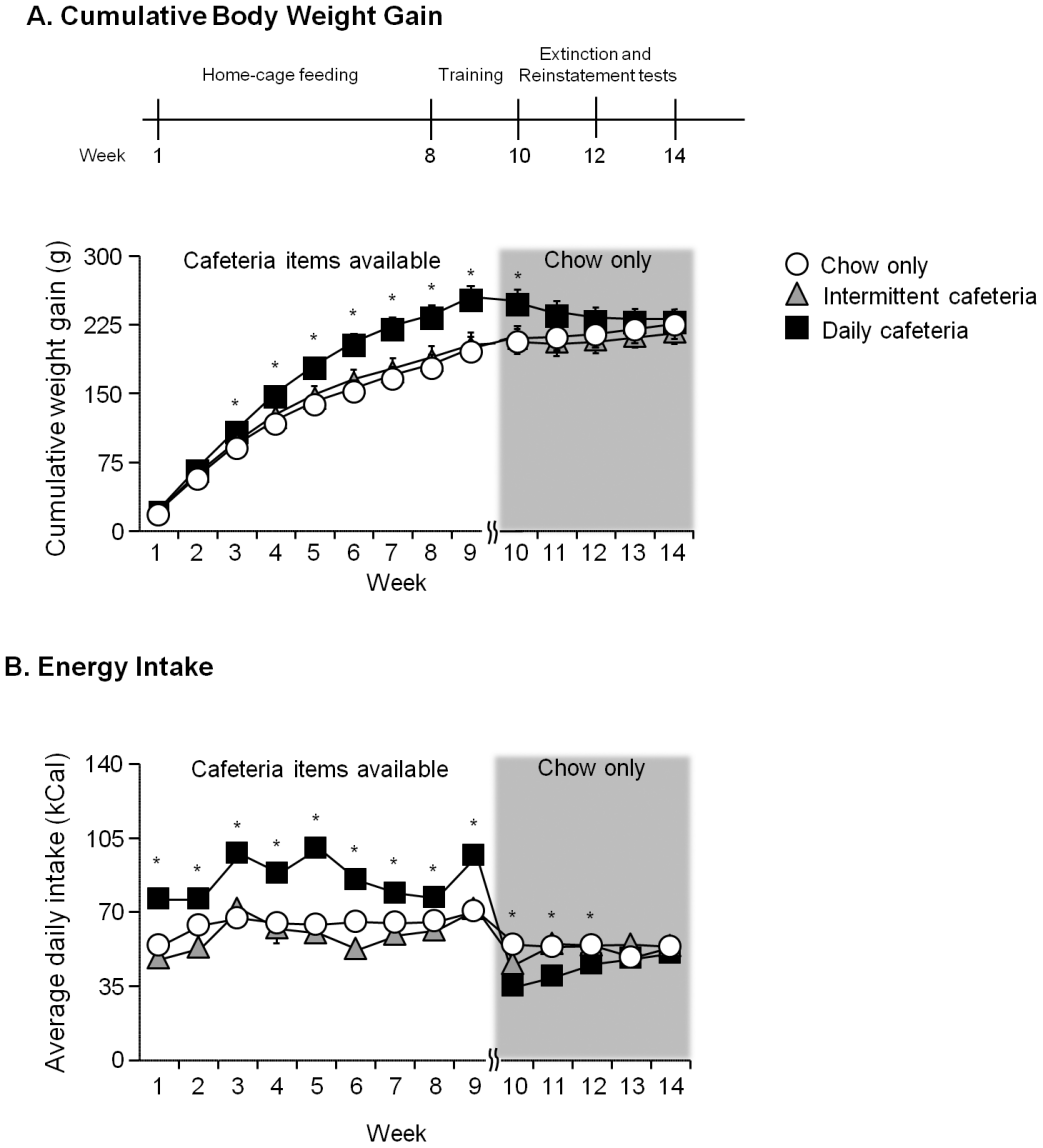
Cumulative body weight gain and average daily caloric intake in rats with different diet conditions. **A.** Weekly average cumulative body weight gain (mean±SEM) in rats with daily access to a cafeteria diet, intermittent access to a cafeteria diet or daily access to chow-only. Weeks 1–7 correspond to the home-cage access phase and Weeks 8–9 correspond to training phase in which access to home-cage diets remained unchanged. Extinction started on Week 10, from which time the cafeteria items were no longer available. All rats had *ad libitum* chow from Week 10 to the end of testing. Discrete-cue induced reinstatement test occurred in Week 12; pellet-priming- and intermittent footshock-induced reinstatement tests occurred in Week 14. **B.** Daily average caloric intake from diet (mean±SEM). *Different between chow-only and daily cafeteria group, p<0.05, n = 8 per group.

Rats that had daily access to cafeteria items gained significantly more body weight and consumed more calories during the diet exposure, but this difference in body weight disappeared once the diet was no longer available. From PND 32 all rats had home-cage access to their designated diet (daily cafeteria, intermittent cafeteria, chow only) for 64 days before cafeteria diets were removed and extinction began. The cumulative body weight gain and caloric intake during these phases are shown in **Fig. 1**. For the body weight gain during the first phase, when the cafeteria items were available in home-cages (weeks 1 to 9), we analyzed the data using the mixed ANOVA, with the between-subjects factor of Diet Condition and within-subjects factor of Time (weeks 1 to 9). Statistical analysis on cumulative body weight gain showed significant main effects of Diet Condition and Time (F_2,21_ = 6.8 and F_8,168_ = 839.0, respectively, p<0.01), and a significant interaction of Diet Condition x Time (F_16,168_ = 8.5, p<0.01). Post-hoc analysis, as depicted in **Fig. 1A**, showed that the daily cafeteria group gained significantly more weight than the chow-only group from week 3 to the end of the diet exposure period. Similarly, analysis of caloric intake showed significant main effects of Diet Condition and Time (F_2,21_ = 39.5 and F_8,168_ = 24.5, respectively, p<0.01), and a significant interaction of Diet Condition x Time (F_16,168_ = 4.8, p<0.01). Post-hoc analysis (**Fig. 1B**) showed that the daily cafeteria group had significantly more caloric intake than chow-only groups from week 1.

Once the cafeteria items were removed (weeks 10–14), a similar analysis on cumulative body weight gain as stated above showed a significant main effect of Time (F_4,84_ = 7.9, p<0.01) and interaction of Diet Condition x Time (F_8,64_ = 17.6, p<0.01), but not Diet Condition (p = 0.29), suggesting that there was no longer a body weight difference between groups. Post-hoc analysis (**Fig. 1A** **grey shaded region**) showed that the daily cafeteria group weighed significantly more only during the first week following cessation of cafeteria diet, and there were no group differences during reinstatement testing. A similar analysis on caloric intake after the cessation of cafeteria diet as stated above showed significant main effects of Diet Condition and Time (F_2,21_ = 9.0 and F_4,84_ = 6.3, respectively, p<0.01) and interaction of Diet Condition x Time (F_8,84_ = 4.7, p<0.01). Post-hoc analysis (**Fig. 1B** **grey shaded region**) showed that the daily cafeteria group had significantly less caloric intake than the chow-only group for the first three weeks following cessation of cafeteria diet.

### Effect of cafeteria diet history on pellet self-administration and extinction

#### Training

**Figure 2:**
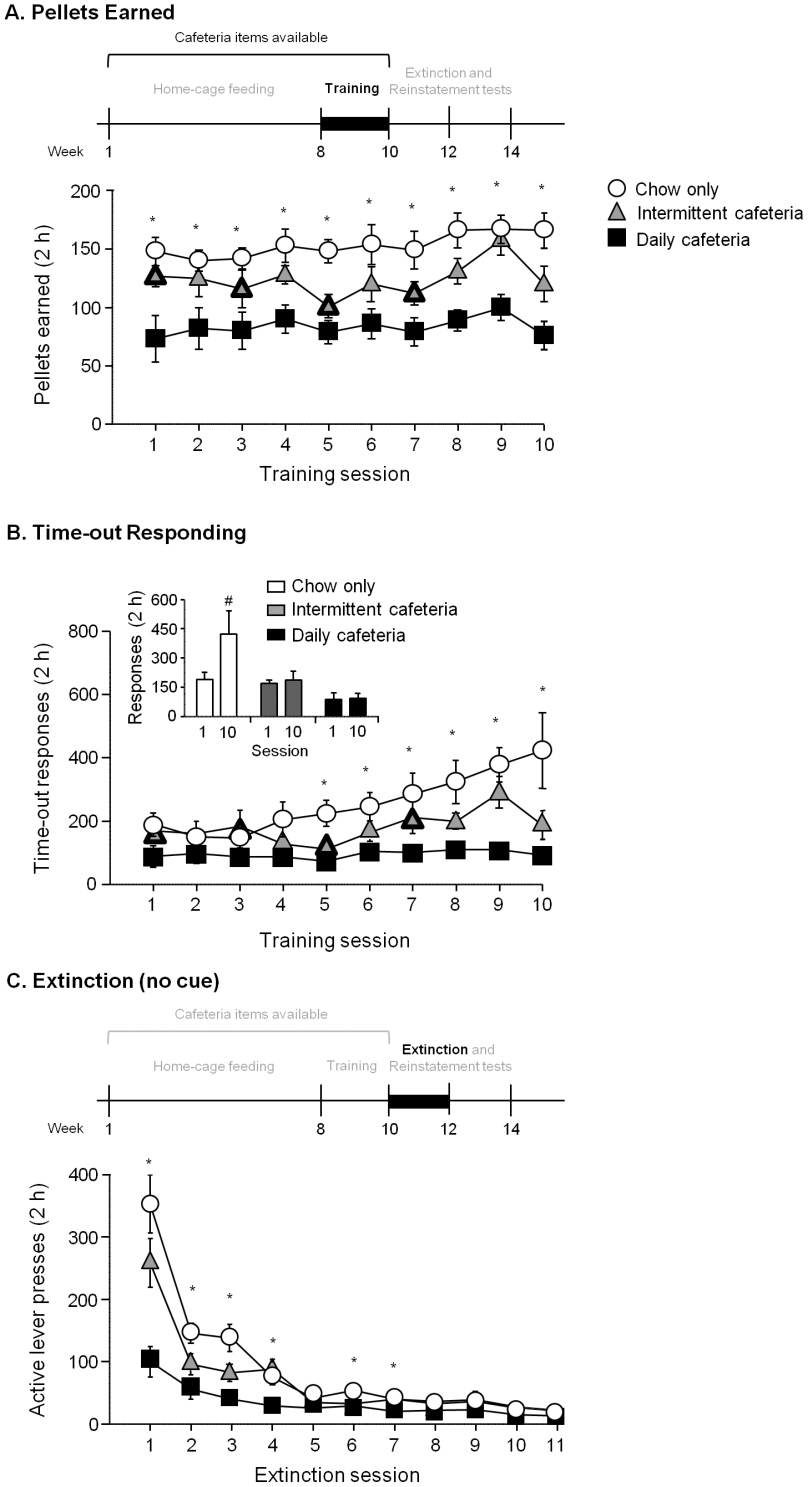
Effect of home-cage diet condition on food self-administration training. **A.** Reinforcers (pellets) earned (mean±SEM). **B.** Time-out responding during food self-administration training for different diet conditions. Inset: difference in time-out between training sessions 1 and 10 for each diet condition. ^#^ Different between sessions 1 and 10 within diet condition, p<0.05. **C.** Active lever responding (mean±SEM) under extinction conditions without cue (lever responding had no consequence).*Different between chow-only and daily cafeteria group, p<0.05, n = 8 per group. Data points with heavier outlines indicate days that intermittent group had access to cafeteria items prior to the session.

All rats demonstrated reliable food-reinforced responding, but the magnitude and time course of responding over days differed between diet conditions with significantly lower food self-administration in rats in the daily cafeteria diet condition (**Fig. 2**). We analyzed the data using the mixed ANOVA, with the between-subjects factor of Diet Condition and within-subjects factor of Session. Analyses of pellets earned showed a significant main effect of Diet Condition and Session (F_2,21_ = 16.6 and F_9,189_ = 2.3, respectively, p<0.05), but no significant interaction of Diet Condition x Session (p = 0.96). Post-hoc differences are depicted in **Fig. 2A**. Analyses on time-out responses on the active lever showed a significant main effect of Diet Condition and Session (F_2,21_ = 9.1 and F_9,189_ = 5.0, respectively, p<0.01), and a significant interaction of Diet Condition x Session (F_18,189_ = 2.1, p<0.01). A post-hoc analysis comparing the time-out responses between sessions 1 and 10 for each diet condition showed that the chow-only group, but not the daily or intermittent cafeteria groups, demonstrated escalated time-out responding after 10 days of training (t_7_ = 2.4, p<0.05) (**Fig. 2B** **, inset**). Additional analysis showed that while the intermittent group had access to cafeteria items before some training sessions, this did not affect their time-out responding (cafeteria diet available: 168.5±21.0 vs. cafeteria diet not available: 188.0±22.9). Inactive lever pressing data for the training phase are presented in table 1.

**Table 1:** Summary of inactive-lever presses during the training, extinction, and reinstatement testing.

		Chow only	Intermittent cafeteria	Daily cafeteria
Training	day 1	20.3±7.9	22.8±7.1	11.6±7.4
	day 10	10.5±4.8	3.4±1.1	2.5±1.1
Extinction (no cue)	day 1	19.6±8.7	6.3±2.9	4.1±2.3
	day 10	4.4±1.5	7.9±3.5	2.9±1.8
Cue-induced reinstatement	No cue	4.4±1.7	8.5±4.0	16.0±14.9
	Cue	9.1±3.5	6.6±3.4	2.4±2.0
Pellet priming-induced reinstatement	0 pellet	2.8±1.2	1.8±0.9	0.8±0.5
	1 pellet	2.9±1.7	2.5±1.7	1.6±1.2
	2 pellets	1.4±0.7	2.4±1.3	5.6±4.6
	4 pellets	4.6±2.3	1.3±0.6	9.8±7.0
Footshock-induced reinstatement	0 min	3.0±1.1	3.6±1.9	5.3±3.0
	5 min	7.5±2.7	4.3±1.6	8.8±6.0
	10 min	4.6±2.3	13.4±10.8	6.6±2.9

Data are mean±SEM.

#### Extinction

All rats reduced active lever responding during the extinction phase, both in the absence and presence of the tone-light cue (prior to or after cue-induced reinstatement, respectively), but similar to food self-administration lever responding was significantly lower in the daily cafeteria group (**Fig. 2C**). The analysis using the mixed ANOVA, with the between-subjects factor of Diet Condition, and within-subjects factors of Lever (active, inactive) and Session, showed significant main effects of Diet Condition and Session (F_2,21_ = 8.7 and F_10,210_ = 72.3, p<0.01) and interactions of Diet Condition x Lever, Diet Condition x Session, and Lever x Session (F_2,21_ = 8.5, F_20,210_ = 9.0, and F_10,210_ = 62.2, respectively, p<0.01), and Diet Condition x Lever x Session (F_20,210_ = 6.8, p<0.01). Post-hoc differences between groups were depicted in **Fig. 2C**, and consistent with the level of lever pressing observed during training, chow-only had greater lever responding compared to the daily cafeteria during the early extinction sessions. Inactive lever pressing data for the extinction phase are presented in table 1.

### Effects of cafeteria diet history on cue-induced reinstatement

**Figure 3:**
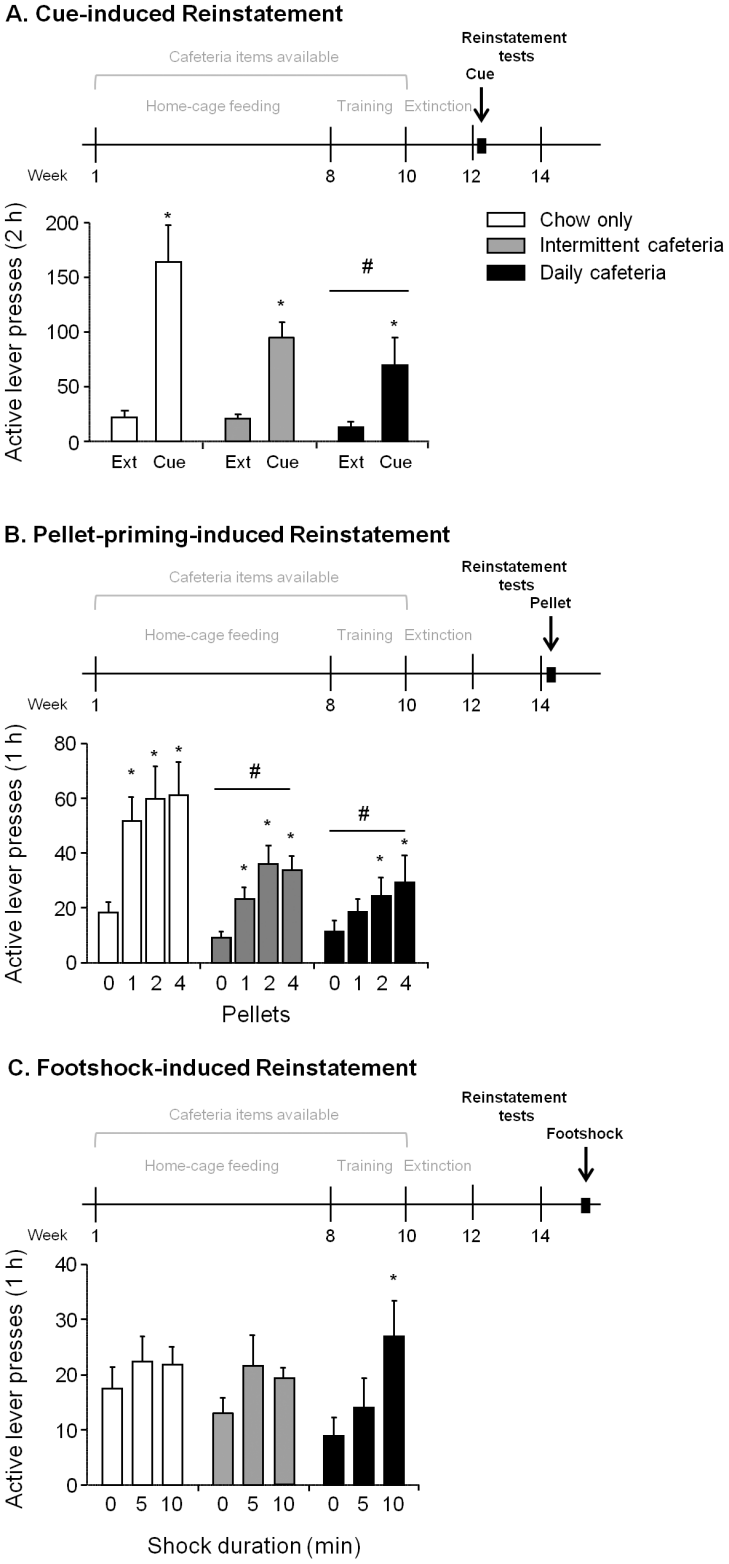
Effect of home-cage diet histories on reinstatement of food seeking. **A.** Effect of discrete cue presentation on reinstatement. Active lever responding (mean±SEM) under extinction (with cue) conditions following a single non-contingent cue presentation at the start of the session. *Different between the last day of extinction (without cue) and cue-test within subject, p<0.05. ^#^ Different in overall lever pressing from chow-only group, p = 0.06. **B.** Effect of pellet priming on reinstatement. Active lever responding (mean±SEM) under extinction conditions (with cue) following 1, 2, and 4 non-contingent pellet deliveries at the beginning of an extinction session. *Different between 0 pellet baseline (last one hour extinction session) and respective pellet test within subject, p<0.05. ^#^ Different in overall lever pressing from chow-only group, p<0.05. **C.** Effect of intermittent footshock on reinstatement. Active lever responding (mean±SEM) under extinction conditions (with cue) following 5 min and 10 min of intermittent footshock.

Discrete tone-light cues successfully reinstated active lever responding in all rats, but the magnitude of responding was lower in rats with a history of cafeteria diet (**Fig. 3A**). Data were analyzed using mixed-factor ANOVA, with the between-subjects factor of Diet Condition, within-subjects factor of Lever (active, inactive) and Reinstatement Condition (last day extinction [no cue], cue). We found significant main effects of Lever and Reinstatement Condition (F_1,21_ = 62.6 and F_1,21_ = 45.0, respectively, p<0.01), but not Diet Condition (p = 0.15), and significant interactions of Diet Condition x Lever, Diet Condition x Reinstatement Condition, and Lever x Reinstatement Condition (F_2,21_ = 4.9, F_2,21_ = 5.5, F_1,21_ = 35.4, p<0.05). A post-hoc comparison showed an approaching significant difference between lever responding in the chow-only compared to the daily cafeteria group (p = 0.06). Overall, our results showed that while the cue-induced reinstatement effect is observed in all groups, this effect is most pronounced in the chow-only group and least pronounced in the daily cafeteria group.

### Effects of cafeteria diet history on pellet priming-induced reinstatement

Similar to the cue-induced reinstatement test, pellet-priming-induced reinstatement was more pronounced in the chow-only group than in the rats with a history of cafeteria diet (**Fig. 3B**). Data were analyzed using mixed-factor ANOVA, with the between-subjects factor of Diet Condition, within-subjects factors of Lever (active, inactive) and Pellet Number (0, 1, 2, 4). The results showed significant main effects of Diet Condition, Lever, and Pellet Number (F_2,21_ = 4.1, F_1,21_ = 79.1, and F_3,63_ = 16.5, respectively, p<0.05), as well as significant interactions of Diet Condition x Lever, Lever x Pellet Number, and Diet Condition x Lever x Pellet Number (F_2,21_ = 7.2, F_3,63_ = 18.6, F_6,63_ = 2.9, p<0.05). Lever responding across all pellet priming conditions in the chow-only group was significantly higher than either intermittent cafeteria or daily cafeteria groups (p<0.05). In order to further understand the effect of Pellet Number on groups with cafeteria diet histories, we performed three separate repeated measures ANOVAs for chow-only, daily cafeteria and intermittent cafeteria groups, using the within-subjects factors of Lever (active, inactive) and Pellet Number (0, 1, 2, 4). Results from the analyses of daily and intermittent cafeteria diet groups showed significant main effects of Lever and Pellet Number (daily cafeteria: F_1,7_ = 16.1, F_3,21_ = 3.3, respectively, p<0.05; intermittent cafeteria: F_1,7_ = 67.5, F_3,21_ = 5.9, respectively, p<0.01), and interaction of Lever x Pellet Number (F_3,21_ = 9.9, p<0.01) in the intermittent cafeteria group only. Thus, similar to the effect of discrete cue, the reinstatement induced by pellet priming was most robust in chow-only and least robust in the daily cafeteria rats.

### Effects of cafeteria diet history on intermittent footshock-induced reinstatement

Intermittent footshock induced modest reinstatement of lever pressing; this effect was significant in the daily cafeteria group, but not the intermittent cafeteria or chow-only groups (**Fig. 3C**). Data were analyzed using mixed-factor ANOVA, with the between-subjects factor of Diet Condition, within-subjects factor of Lever (active, inactive) and Shock Duration (0, 5, and 10 min). The results showed significant main effects of Lever and Shock Duration (F_1,21_ = 39.0, F_2,42_ = 4.0, p<0.05), but not Diet Condition (p = 0.93). In order to further understand the main effect of shock across groups, we performed two separate repeated measures ANOVAs, with Lever (active, inactive) and Shock Duration (0, 5, and 10 min) as within-subjects factors, focusing on rats that previously had access to cafeteria diets. The analysis of the daily cafeteria group only showed a Lever x Shock Duration interaction (F_2,14_ = 3.7, p = 0.05), and post-hoc tests on active lever responding showed a significant difference between 0 min and 10 min shock (p<0.05). A similar analysis of the intermittent cafeteria group failed to show a significant interaction. Thus, in contrast to discrete cue or pellet-priming, the daily cafeteria diet group appears to be more sensitive to intermittent footshock-induced reinstatement than the intermittent cafeteria or chow-only groups.

## Discussion

In the present study, we examined reinstatement of food seeking in a more clinically relevant rat model, which used unrestricted female rats that became overweight by prolonged exposure to a cafeteria diet. In agreement with previous reports [34], [35], we found that daily exposure to cafeteria diet leads to increased caloric intake and significant weight gain. This diet manipulation, however, led to decreased concurrent self-administration of normally preferred food pellets. After cessation of diet exposure in the home-cage and removal of the preferred food pellets from the operant chambers during the extinction and reinstatement phases, rats with prior history of daily cafeteria diet, and to a lesser degree prior history of intermittent cafeteria diet, showed decreased extinction responding, and cue- and pellet-priming-induced reinstatement. In contrast, modest stress-induced reinstatement was only observed in rats with a history of daily cafeteria diet. Another finding in our study is that, independent of the history of home-cage diet, female rats with free access to standard chow not only demonstrated escalation in time-out responding during pellet self-administration, but also demonstrated robust cue- and pellet-priming-induced reinstatement. The increase in time-out responding is interesting in light of studies using drug or food reinforcers, which have suggested that lever pressing during the non-reinforced period reflects a compulsive reward seeking behavior that may predict the “addiction-prone” phenotype [36], [37]. These data extend previous reinstatement studies in food-restricted male or female rats during the extinction and reinstatement phases [15], [16] by examining these behaviors in unrestricted female rats.

### Female rats with *ad libitum* access to cafeteria diet alongside standard chow show clinically relevant food intake patterns

The present home-cage food access procedures may provide a more clinically relevant picture of food intake prior to and during dieting than is typical of food reinstatement studies [15]. Initially, humans with unhealthy eating habits consume limited amounts of nutritionally balanced foods and greater amounts of calorically-dense palatable foods. Then during dieting human subjects not only restrict the amount of calorically-dense food they eat but also tend to consume nutritionally balanced foods. Traditional home-cage food restriction in reinstatement studies is an experimenter-administered manipulation that forces the rat to consume less nutritionally balanced food in order to accommodate for palatable food consumption. The current procedures (chow and cafeteria diet freely available) allowed the rats to choose how much nutritionally balanced and calorically-dense food to consume. Under these conditions, rats consumed less nutritionally balanced food and more palatable food (in the home-cage), a pattern similar to the unhealthy eating habits observed in humans prior to dieting. Then during extinction and reinstatement, rats fed cafeteria diets were switched to a chow-only diet, a qualitative manipulation that more closely models human dieting than the maintenance of chow restriction commonly used in reinstatement studies.

The current procedures may prove valuable in revealing the involvement of neural mechanisms in reinstatement that are qualitatively different in overweight rats as compared to normal weight rats. Studies show that chronic access to a cafeteria diet leads to metabolic and immune dysfunctions commonly associated with human obesity [34], [38]. Furthermore, unhealthy diet histories cause dysregulations in reward processing and stress systems [20], [21], both of which are critical in controlling relapse behavior [16], [23].

### Female rats with cafeteria diet history show modest cue- and pellet-priming-induced reinstatement

We found that rats with prior daily and intermittent cafeteria diet history showed relatively modest discrete cue- and pellet-priming-induced reinstatement effects as compared to rats fed with chow only. This is perhaps not surprising, since both cafeteria groups demonstrated low levels of operant pellet self-administration during the training and extinction phases. These effects may be explained by the successive negative contrast effect, which describes a change in animals' behavior following a downshift in the qualitative or quantitative value of an expected reward [39] and is often observed in the context of instrumental settings when food pellets are used as reward [40], [41]. Indeed, since rats compare the present reward with their previous experiences and respond according to its relative value [42], rats that previously had access to highly palatable cafeteria items may not perceive the operant pellet to be as rewarding as the rats fed chow-only.

If the value of outcome is lower for rats with cafeteria diet histories, this might explain the reduced motivation during self-administration and pellet-priming reinstatement testing. Previous work has shown that rats with access to sucrose during adolescence show a lower motivation to self-administer saccharin during adulthood [43]. Further, this less effective reinforcer would presumably support less incentive salience attribution to the pellet-associated cues [44], which may reduce the cue's effectiveness at reinstating behavior. The possible influence of such negative contrast effects in blunting motivation in the operant setting should be carefully considered in studies that seek to make rats overweight using palatable diets prior to assessing motivation for other natural and drug rewards.

Exposure to the daily cafeteria diet may also lead to a depressive-like, anhedonic state, which could contribute to the lack of responding during reinstatement tests observed in cafeteria rats. Exposure to energy dense diets elevates brain stimulation reward threshold in rats [35], and also increases depression-related behaviors in forced swim and sucrose preference tests in mice [45], [46]. Withdrawal from palatable food, as experienced by the daily cafeteria rats during the extinction and testing phases of the present study, also leads to depressive-like behaviors in rats using similar diet manipulations [47]. Other studies have found that naltrexone and social defeat, both of which cause anhedonia [48], [49], either suppress responding in reinstatement testing [50] or reduce lever responding during extinction [51]. Thus, an anhedonic state associated with withdrawal from cafeteria diets may contribute to the blunted reinstatement effects observed in the present study, another important consideration for future studies.

### Female rats with prior cafeteria diet history show modest stress-induced reinstatement

Although rats that previously had daily access to cafeteria diet consistently showed less lever responding during training, extinction and cue- and pellet-priming-induced reinstatement, they were particularly sensitive to stress-induced reinstatement. Indeed, while chow-only rats did not respond to footshock-induced reinstatement of food seeking, a pattern similar to previous findings [52], [53], the daily cafeteria group significantly increased lever pressing after 10 min of intermittent footshock. This heightened sensitivity to stress is consistent with studies in rodents showing that extended access to and withdrawal from palatable food causes adaptations in stress circuitry [22], [54]–[57]. In the present study, rats previously fed cafeteria diet, which restrict intake of chow once palatable food is no longer available, show greater sensitivity to stress-induced reinstatement, a finding that parallels the clinical observation that restraint eaters have a greater tendency to overeat when encountering a stressful situation [58], [59]. Future studies are needed to determine the underlying mechanisms and related physiological changes of this diet-induced hypersensitivity to stress. For example, while studies have shown that access to or removal of cafeteria diet did not affect baseline corticosterone levels [56], diet history can affect how stress circuitry responds to acute stressors [60].

The stress-effect in the cafeteria group should, however, be interpreted with caution. First, the amount of lever pressing observed in the cafeteria group after footshock, while significantly different than baseline responding, is not as robust as the cue and pellet priming reinstatement effects reported here and footshock stress effect reported previously with heroin and cocaine [31], [32], [61]. Second, all rats were repeatedly tested across multiple reinstating stimuli, and footshock-induced reinstatement testing was conducted last. However, repeated testing is unlikely to confound our interpretations, since all rats underwent additional extinction sessions before footshock testing, and the baseline lever-pressing for the cafeteria group remained consistent across all reinstatement testing. Similar repeated testing procedures have also been used in various reinstatement studies and reliable results are obtained [25], [62]–[68].

Finally, footshock-induced reinstatement testing occurred 4–5 weeks after the cessation of cafeteria diets. At the time of footshock testing both body weight and caloric intake differences between cafeteria and chow-only groups had disappeared. When examining the impact of historical manipulations such as exposure to or withdrawal from cafeteria diets, it can be argued that it would be ideal to do so at an earlier time point than that used in the present study. However, similar withdrawal periods have been used in drug studies where footshock-induced reinstatement effects were found [31], [32], [61]. Further, the stress sensitivity observed here could be time-dependent, as is the incubation of drug or sucrose craving [69]. Future studies are needed to determine the time-course of this potentially enhanced sensitivity to stress in cafeteria rats.

## Conclusions

Here, we demonstrate that it is possible to study reinstatement of food seeking in a clinically relevant model that makes rats overweight by giving them access to unhealthy foods commonly consumed by humans. Female rats serve as suitable subjects for reinstatement studies, as under the current procedures they demonstrated robust self-administration responding and reinstatement effects without the need for food restriction. Studies have shown that chronic caloric restriction can cause long-lasting changes in the stress- and feeding-related pathways [54], and the level of food restriction may contribute to the differential effect of pharmacological agents on reinstatement of food seeking [15]. A potential concern when using intact female rats is that fluctuations in ovarian hormones across the estrous cycle can influence reinstatement behavior [64], [70], [71]. In the present study we did not directly track estrous cycle phase, and thus we cannot rule out the possibility that cafeteria diet histories influence the estrous cycle, which might contribute to observed differences in reinstatement behavior between groups. Nevertheless, a previous study has shown that ovarian hormones and estrous cycle phase play relatively insignificant roles in reinstatement of palatable food seeking [72]. Together with a recent study showing that the behavior of female rodents is no more intrinsically variable than that of male rodents, as commonly assumed [73], our results provide a useful procedural validation for future studies that intend to study reinstatement under conditions that more accurately model clinical observations in individuals seeking treatment for relapse to unhealthy eating during dieting.
